# Endoscopic treatment of a post-surgical dehiscence of an esophageal–ileal–colonic reconstruction: when endoscopy reaches its limit

**DOI:** 10.1055/a-2088-8491

**Published:** 2023-06-12

**Authors:** Giacomo Emanuele Maria Rizzo, Lucio Carrozza, Salvatore Tammaro, Dario Ligresti, Ilaria Tarantino, Mario Traina

**Affiliations:** 1 Endoscopy Service, Department of Diagnostic and Therapeutic Services, IRCCS – ISMETT, Palermo, Italy; 2 Department of Surgical, Oncological and Oral Sciences (Di.Chir.On.S.), University of Palermo, Palermo, Italy

A 55-year-old man was referred to our institute after total gastrectomy and partial esophagectomy for a gastroesophageal tumor. Esophageal reconstruction included the right colon and the last loop of the ileum with esophageal–ileal, colonic–jejunal, and jejuno–jejunal anastomoses (Roux-en-Y reconstruction). Following the initial surgery, a computed tomography scan showed a suspected anastomotic leak with the development of intrathoracic/intra-abdominal collections, so the patient underwent a second surgical procedure with placement of a thoracic/abdominal drain.

The patient’s condition subsequently worsened and he was referred to our institute, where gastroscopy showed a wide (approximately 280°) dehiscence of the esophageal-ileal anastomosis at 20 cm from the oral cavity, with fluoroscopic examination showing extraluminal diffusion of contrast medium into the mediastinum, which was filled with purulent/oily secretions ( Fig. 1 a ). The residual colon was identified after crossing about 20 cm of mediastinum and its mucosa was trophic ( Fig. 1 b ). The colonic–jejunal anastomosis appeared normal with no evidence of any leaks but, as the patient was unwell, we decided to deploy an enteral fully covered self-expandable metal stent (FCSEMS; 150 × 22 mm), with the proximal side fixed in the cervical esophagus ( Fig. 1 c ; Video 1 ). The stent was short and could not create a direct connection within the colon, so a further enteral FCSEMS (80 × 22 mm) was coaxially deployed between the distal portion of the upper FCSEMS and the colonic segment. The overlapping sections were fixed with three metal clips, and fluoroscopy showed no extraluminal diffusion of contrast medium ( Fig. 2 ). Finally, we placed a nasogastric tube with the distal end beyond the second FCSEMS and attached it to an aspiration system.

**Fig. 1:**
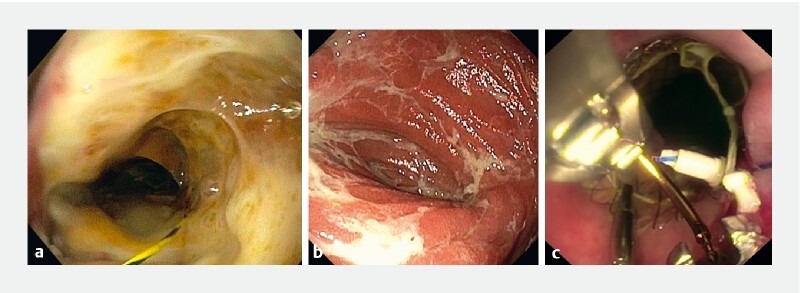
Endoscopic images showing: **a** the anastomotic leak and the development of a purulent framework with abundant oily secretions; **b** the colonic luminal mucosa, which was still trophic, with a mixture of pale and hyperemic areas; **c** the proximal side of the fully covered self-expandable metal stent fixed in the cervical esophagus through a mixture of through-the-scope clips and an endoscopic suture system.

**Video 1** Video showing the endoscopic treatment of a post-surgery dehiscence of an esophageal–ileal–colonic reconstruction performed after surgical resection of a gastroesophageal tumor. The management included double fully covered self-expandable metal stent placement and fixation through endoscopic sutures and metal through-the-scope clips.


**Fig. 2:**
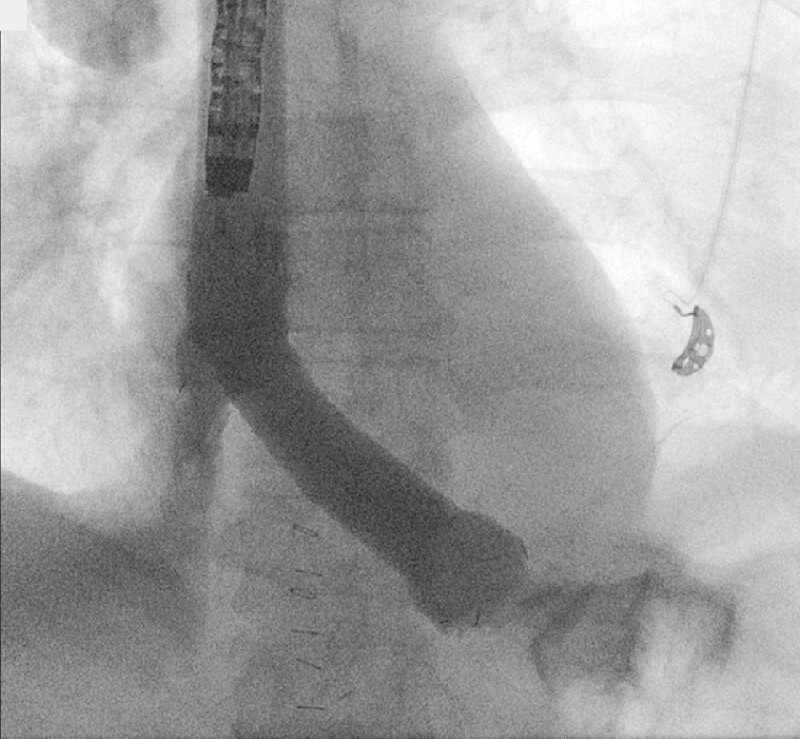
Fluoroscopic view at the end of the procedure showing appropriate flow of contrast through the two stents into the colonic anastomotic lumen and no extraluminal diffusion.

The patient was clinically stable for 3 days and imaging showed the assembled system remained in place ( Fig. 3 ). Unfortunately, the anastomotic colon then became necrotic, so the patient needed to undergo further surgery. Our endoscopic treatment did not achieve complete clinical success, becoming instead a bridge-to-surgery that allowed us to choose the best window for the patient’s next surgical intervention.

**Fig. 3:**
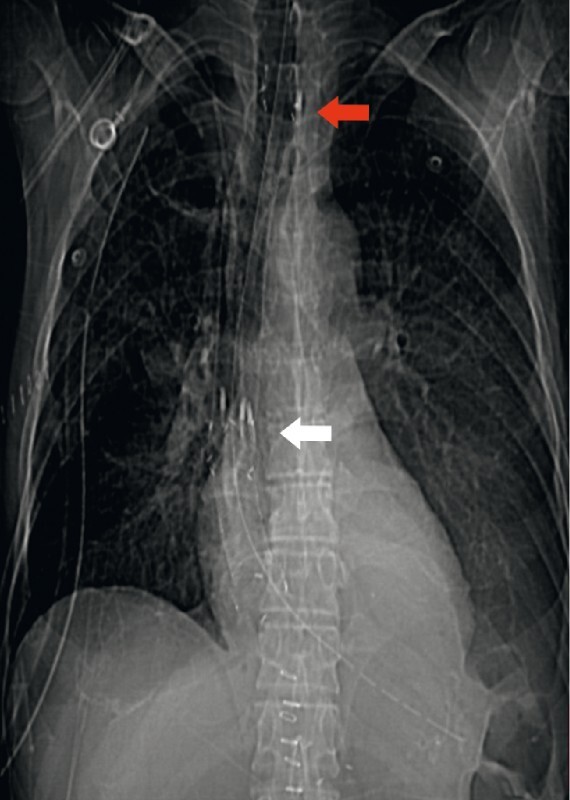
Radiographic image showing the two correctly located stents, with the upper stent fixed at its cranial end in the cervical esophagus (red arrow) and the two stents secured together with clips (white arrow).

Endoscopy_UCTN_Code_TTT_1AQ_2AG

## Notes

Endoscopy E-Videos

https://eref.thieme.de/e-videos
*E-Videos* is an open access online section of the journal *Endoscopy* , reporting on interesting cases and new techniques in gastroenterological endoscopy. All papers include a high-quality video and are published with a Creative Commons CC-BY license. Endoscopy E-Videos qualify for HINARI discounts and waivers and eligibility is automatically checked during the submission process. We grant 100% waivers to articles whose corresponding authors are based in Group A countries and 50% waivers to those who are based in Group B countries as classified by Research4Life (see: https://www.research4life.org/access/eligibility/ ). This section has its own submission website at https://mc.manuscriptcentral.com/e-videos

